# Importance of the identification of *Segniliparus* species from pulmonary infection

**DOI:** 10.1016/j.nmni.2018.05.002

**Published:** 2018-05-25

**Authors:** M. Keikha

**Affiliations:** Department of Microbiology, School of Medicine, Isfahan University of Medical Sciences, Isfahan, Iran

*Actinomycetes* are a broad group of bacteria which contain mycolic acid in the cell-wall structure and is classified as a Gram-positive bacteria with high G + C DNA percentage and peptidoglycan type IV (containing meso-diaminopimelic acid, arabinose and galactose) [1]. Aerobic actinomycetes bacteria consist of several pathogenic species such as the *Mycobacterium tuberculosis* complex, *Mycobacterium leprae,* nontuberculous mycobacteria (NTM), *Nocardia, Gordonia, Rhodococcus, Tsukamurella, Williamsia, Dietzia, Skermania* and *Segniliparus* spp., which can enter the human body through inhalation and traumatic inclusion and which can cause pulmonary, cutaneous, eye, central nervous system, lymphatic, disseminated and catheter-related or shunt infection in immunodeficient patients and in healthy individuals [2], [3]. Among this genera, the genus *Segniliparus* was described by Butler et al. [4] from the sputum of a patient suspected of having tuberculosis in the United States.

Because there is limited information about this genus of bacteria, the aim of the present study was therefore to search online literature and electronic databases for collection of key information about *Segniliparus* spp. in order to compile diagnostic notes about this group of bacteria.

The genus of *Segniliparus* was distinguished from other *Actinomycetes* by a unique mycolic acid pattern, 16S rRNA gene sequence, 68 to 72 mol% DNA G + C percentage and DNA-DNA hybridization techniques. This group of bacteria consists of two species, *Segniliparus rotundus* and *Segniliparus rugosus* [4]. On the basis of a literature review, *S. rugosus* can be considered as an emerging human pathogen which is isolated from the pulmonary infection of cystic fibrosis, bronchiolitis and pneumonia of immunocompromised patients or even immunocompetent patients; this bacterium is also able to promote immunoresponse and degradation of the NF-κB signaling pathway [5], [6], [7]. *Segniliparus* spp. are rapid growing, nonpigmented, acid-fast bacteria which resemble rapidly growing mycobacteria. This group of bacteria grows on Middlebrook 7H9-11 media, but it appears that *S. rugosus* was more resistant to growth-inhibited materials and decontamination methods (NaOH and *N*-acetyl-l-cysteine). It is capable of growing in MacConkey agar and it tolerates Löwenstein-Jensen and American Thoracic Society (ATS) media [4], [7].

This genus of bacteria was identified by high-performance liquid chromatography, Thin-layer chromatography mycolic acid analysis, Ziehl-Neelsen staining, growth rate, pigment production, colony morphology, arylsulfatase (3 days), growth on Löwenstein-Jensen and ATS with sodium chloride and MacConkey agar, iron uptake, tellurite and nitrate reduction, Tween hydrolysis, growth in lysozyme, catalase activity, urease hydrolysis, hydrolysis of acetamide, adenine, casein, citrate, aesculin, hypoxanthine, tyrosine and xanthine and metabolism of various carbohydrates; but phenotypic identification of *Segniliparus* spp. is expensive, time-consuming, labour-intensive and confusing, whereas molecular methods such as 16S rRNA and Internal transcribed spacer (16S rRNA–23S rRNA flanking region) sequencing, PCR–restriction fragment length polymorphism analysis using *hsp65* and *rpoB,* and DNA-DNA hybridization methods are inexpensive and rapid and can be applied for accurate identification and differentiation of *Segniliparus* at the species level [4], [5], [6], [7].

Antimicrobial susceptibility tests of clinical isolates of *Segniliparus* spp. were performed using broth microdilution according to the recommendation of the ATS and according to National Committee for Clinical Laboratory Standards (NCCLS) guidelines. This group of bacteria have different drug susceptibility patterns, but the ATS and Infectious Disease Society of America guidelines recommended that clarithromycin combined with amikacin, cefoxitin or imipenem for 2 to 4 months frequently have efficient results in the treatment of pulmonary infections like those caused by this bacteria [8], [9].

On the basis of the clinical reports, the pulmonary infection of *Segniliparus* spp. is associated with chronic cough, fever and hypoventilation, as well as the presence of multiple small nodules, with symptoms of acid-fast bacilli in sputum and radiologic results similar to *M. tuberculosis,* NTM, *Nocardia* and so on. Therefore, pulmonary infection should be identified in microbiology laboratories [4], [5], [6], [7], [8].

In summary, the genus *Segniliparus,* and especially *S. rugosus,* has emerged as a human pathogen isolated from pulmonary infections. *Segniliparus* spp. are similar to rapidly growing mycobacteria which can be misidentified as NTM. Conversely, the pulmonary infection of this group of bacteria is similar to tuberculosis and can be misidentified with other related aerobic actinomycetes, although the antibiotic–drug susceptibility patterns of these infections are different. Therefore, *Segniliparus* infection should be identified to permit final diagnosis, appropriate treatment, patient management and epidemiologic studies. Molecular methods can be applied for accurate identification and differentiation of *Segniliparus* spp.

## Conflict of interest

None declared.
